# Supplementary material for: Rhodobacter capsulatus forms a compact crescent-shaped LH1–RC photocomplex
Source: Nat Commun. 2023 Feb 15;14:846. doi: 10.1038/s41467-023-36460-w (PMC9932092; doi:10.1038/s41467-023-36460-w)
Supplement: Supplementary file 1 — Supplementary Information [file 41467_2023_36460_MOESM1_ESM.pdf]

SUPPLEMENTARY INFORMATION

Rhodobacter capsulatus Forms an Unusually Compact Crescent-Shaped LH1–RC Photocomplex

K. Tani, R. Kanno et al.

Supplementary Table 1 Cryo-EM data collection, refinement and validation statistics.

	LH1-RC-PufX complex of *Rba. capsulatus* (EMDB-33931, PDB ID: 7YML)
**Data collection and processing**	
Magnification	96000
Voltage (kV)	300
Electron exposure (e–/Å^2)	40
Defocus range (μm)	–0.7 to –2.8
Pixel size (Å)	0.820
Symmetry imposed	C1
Initial particle images (no.)	297039
Final particle images (no.)	224431
Map resolution (Å)	2.6
FSC threshold	0.143
Map resolution range (Å)	328–2.6
**Refinement**	
Initial model used (PDB code)	7F0L
Model resolution (Å)	2.7
FSC threshold	0.5
Model resolution range (Å)	128–2.6
Map sharpening *B* factor (Å^2)	–50
Model composition	
Non-hydrogen atoms	18555
Protein residues	1848
Ligands	89
Waters	7
B factors (Å^2)	
Protein	53.0
Ligand	59.2
Waters	37.6
R.m.s. deviations	
Bond lengths (Å)	0.007
Bond angles (°)	2.726
Validation	
MolProbity score	1.91
Clashscore	11.63
Poor rotamers (%)	1.88
Ramachandran plot	
Favored (%)	97.38
Allowed (%)	2.62
Disallowed (%)	0.00

Supplementary Table 2 Comparisons of the distances of His–BChl(Mg) and BChl(Mg)–BChl(Mg) in LH1, LH2 and RC special pairs from various phototrophic bacteria.

	Distance of His(Nε2) to BChl–Mg (Å)		Distance of Mg–Mg (Å)		
LH1 or LH2	α	β	Long	Short	PDB ID
Rba. capsulatus	**2.63**	**2.23**	**9.46**	**8.28**	**7YML**
Rba. sphaeroides (Dimer)	2.58	2.21	9.45	8.36	7VY2
Rba. sphaeroides (Monomer)	2.58	2.20	9.55	8.37	7F0L
Rsp. rubrum (LH1)	2.27	2.03	9.34	8.51	7EQD
Rps. palustris (LH1-W)	2.93	2.71	9.61	8.2	6Z5S
Tch. tepidum (LH1)	2.19	2.19	8.88	8.72	5Y5S
Trv. strain 970 (LH1)	2.33	2.31	8.90	8.46	7C9R
Blc. viridis (LH1)	2.54	2.25	8.8	8.5	6ET5
Rfx. castenholzii (B880)	2.32	2.29	9.5	9.3	5YQ7
Rbl. acidophilus (B850)	2.34	2.34	9.5	8.8	1NKZ
Phs. molischianum (B850)	2.27	2.32	9.2	8.9	1LGH

RC (special pair)	L-subunit	M-subunit	BChl *a* (L)–BChl *a* (M)	
Rba. capsulatus	**2.28**	**2.33**	**7.65**	**7YML**
Rba. sphaeroides (LH1–RC)	2.22	2.13	7.85	7VY2
Rba. sphaeroides (RC-only)	2.27	2.06	7.84	2J8C
Rsp. rubrum	2.09	2.12	7.76	7EQD
Rps. palustris	2.73	2.74	7.69	6Z5S
Tch. tepidum	2.17	2.19	7.87	5Y5S
Trv. strain 970	2.55	2.58	7.92	7C9R
Blc. viridis	2.36	2.35	7.83	6ET5

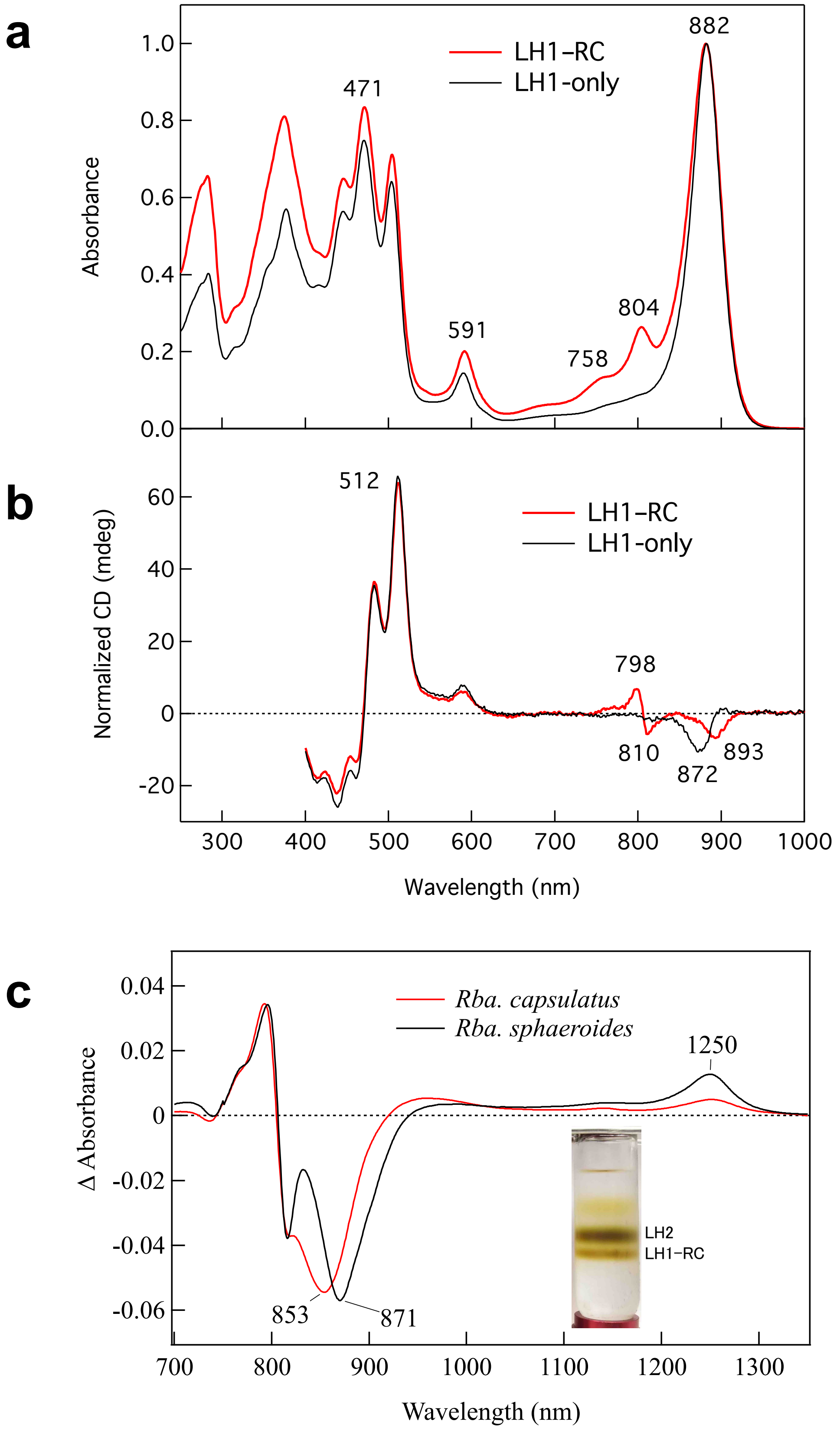

Supplementary Fig. 1 Room temperature absorption and CD spectra of the *Rba. capsulatus* LH1–RC purified by DEAE chromatography. **(a)** Absorption spectra of LH1–RC (red) and LH1-only (black) complexes. **(b)** CD spectra of LH1–RC (red) and LH1-only (black) complexes normalized by corresponding absorption intensities at 882 nm. **(c)** Light-induced P^+/P absorption difference spectra of the LH1–RCs purified from *Rba*. *capsulatus* (red) and *Rba*. *sphaeroides* strain DP2 (black) by subtracting spectra in the dark from those measured during illumination with 940 nm-LED light. Inset shows sucrose density gradient centrifugation (10–40% w/v) of the solubilized *Rba*. *capsulatus* photocomplexes by 1% w/v DDM.

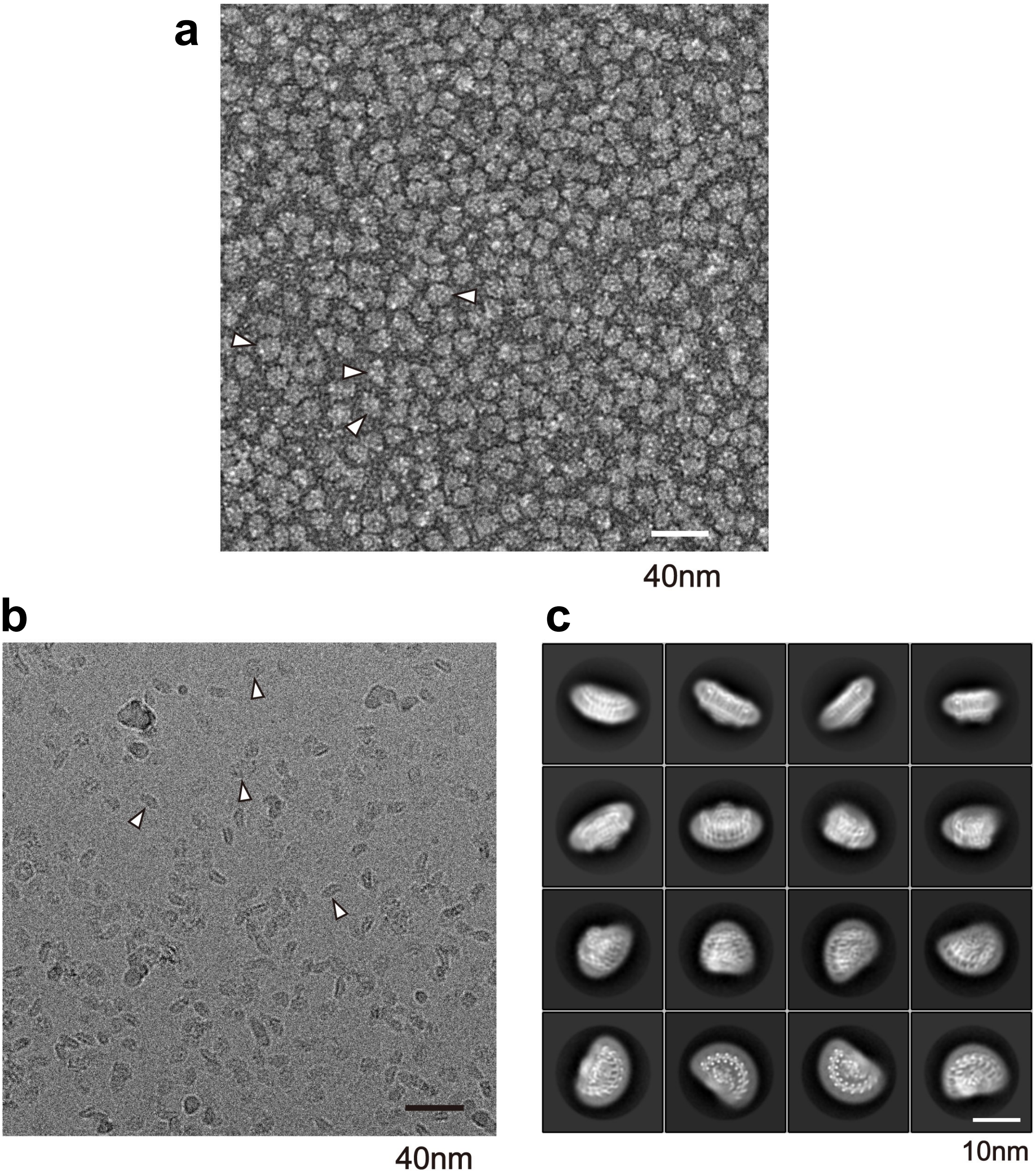

Supplementary Fig. 2 Electron micrographs of the *Rba. capsulatus* LH1–RC complex. **(a)** Negative-stain micrograph of the purified LH1–RC particles that were used for cryo-EM grid preparation. **(b)** Representative cryo-EM micrograph. **(c)** Representative 2D class averages from cryo-EM micrographs. White arrows in **(a)** and **(b)** indicate several representative LH1–RC particles. Data shown in **(a)** and **(b)** were observed from different areas (*N* = 9 and 4343 for **(a)** and **(b)**, respectively) of multiple grids (*N* = 2 and 6 for **(a)** and **(b)**, respectively).

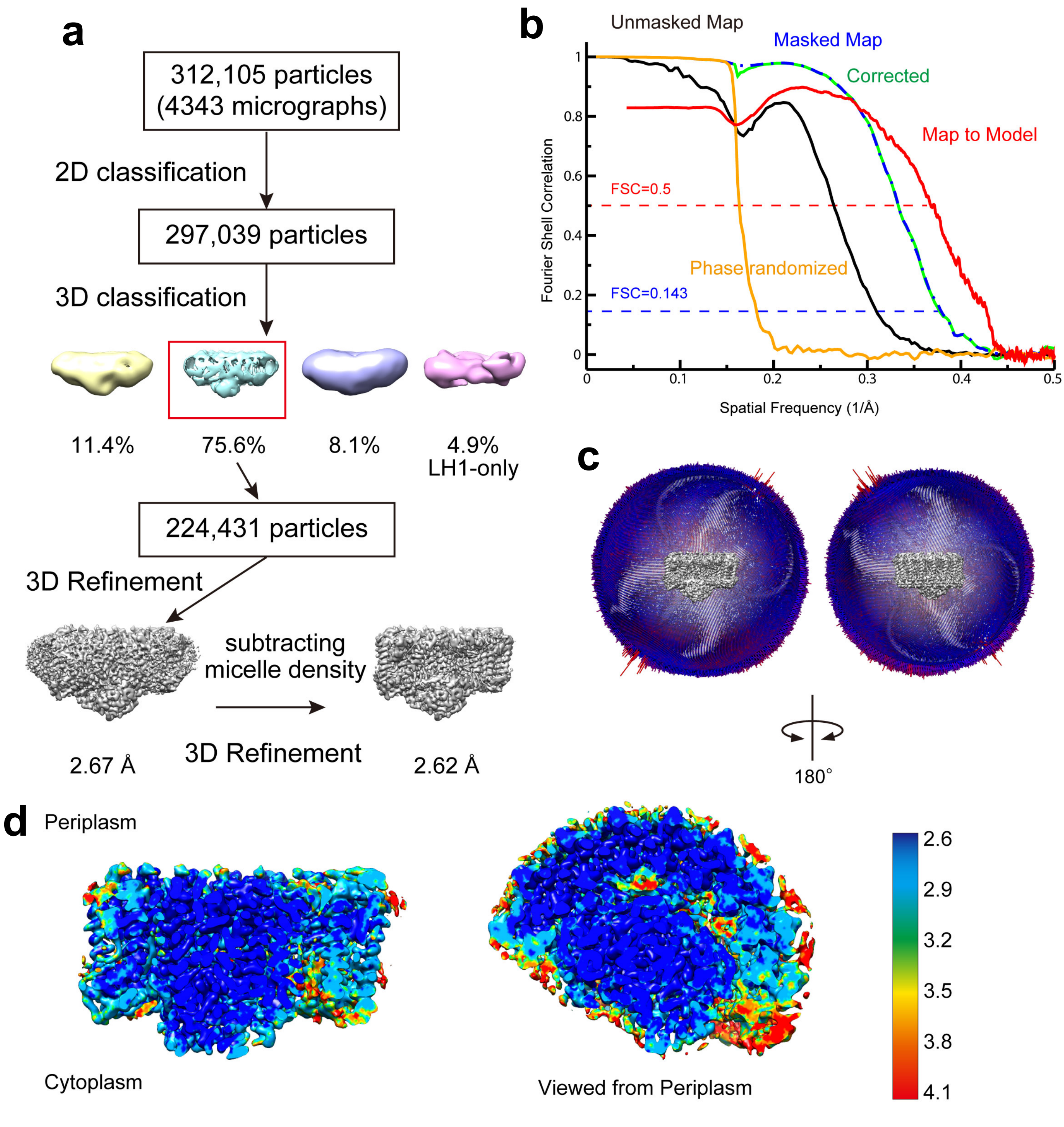

Supplementary Fig. 3 Structure determination of the *Rba. capsulatus* LH1–RC complex by cryo-EM. (a) Image processing flow of 3D classification and reconstruction. **(b)** The Fourier shell correlation (FSC) plots of the cryo-EM map (unmasked: black, masked: blue, phase randomized corrected: green, phase randomized: orange) and the FSC plot of the model versus the final map (red) are superimposed. **(c)** Angular distribution of reconstructed particles. **(d)** Local resolution representation of the LH1–RC structure. A longitudinal sectional view (*left*) and a central cross sectioned view (*right*) from periplasmic side. The map is shown in rainbow colors as shown in the right color bar. The contour level of the map raw density is 0.016.

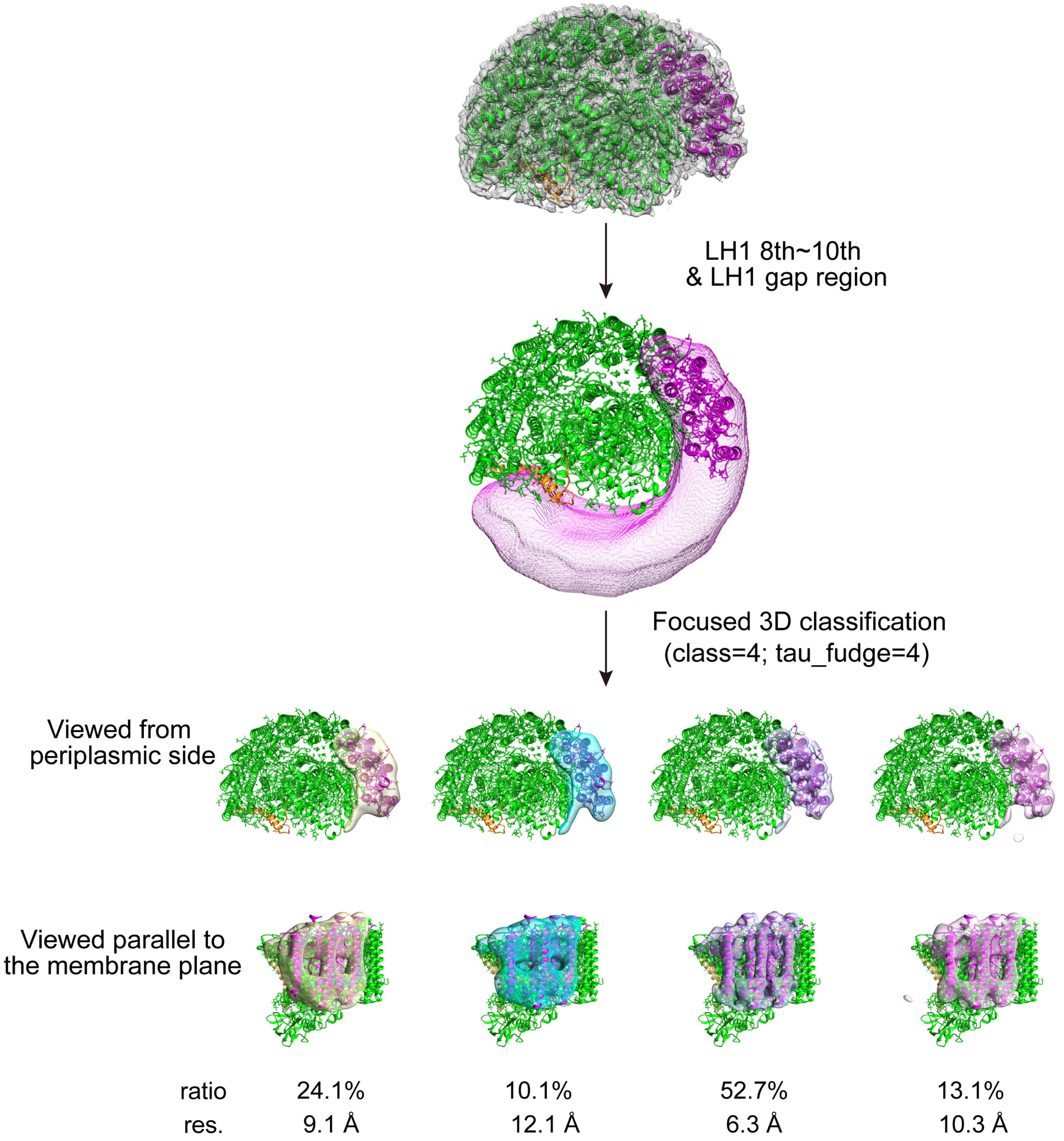

Supplementary Fig. 4 Focused 3D classification of the *Rba. capsulatus* LH1–RC complex. Focused classifications of three LH1-αβ pairs (magenta) into 4 groups, respectively. The numbers below each class indicate resolutions and ratios of the particles belonging to each class. Viewed from periplasmic side except for the lowest row. Density maps for all classes fully cover the three LH1 pairs, and there are no other significant densities corresponding to the LH1 independent of their resolutions.

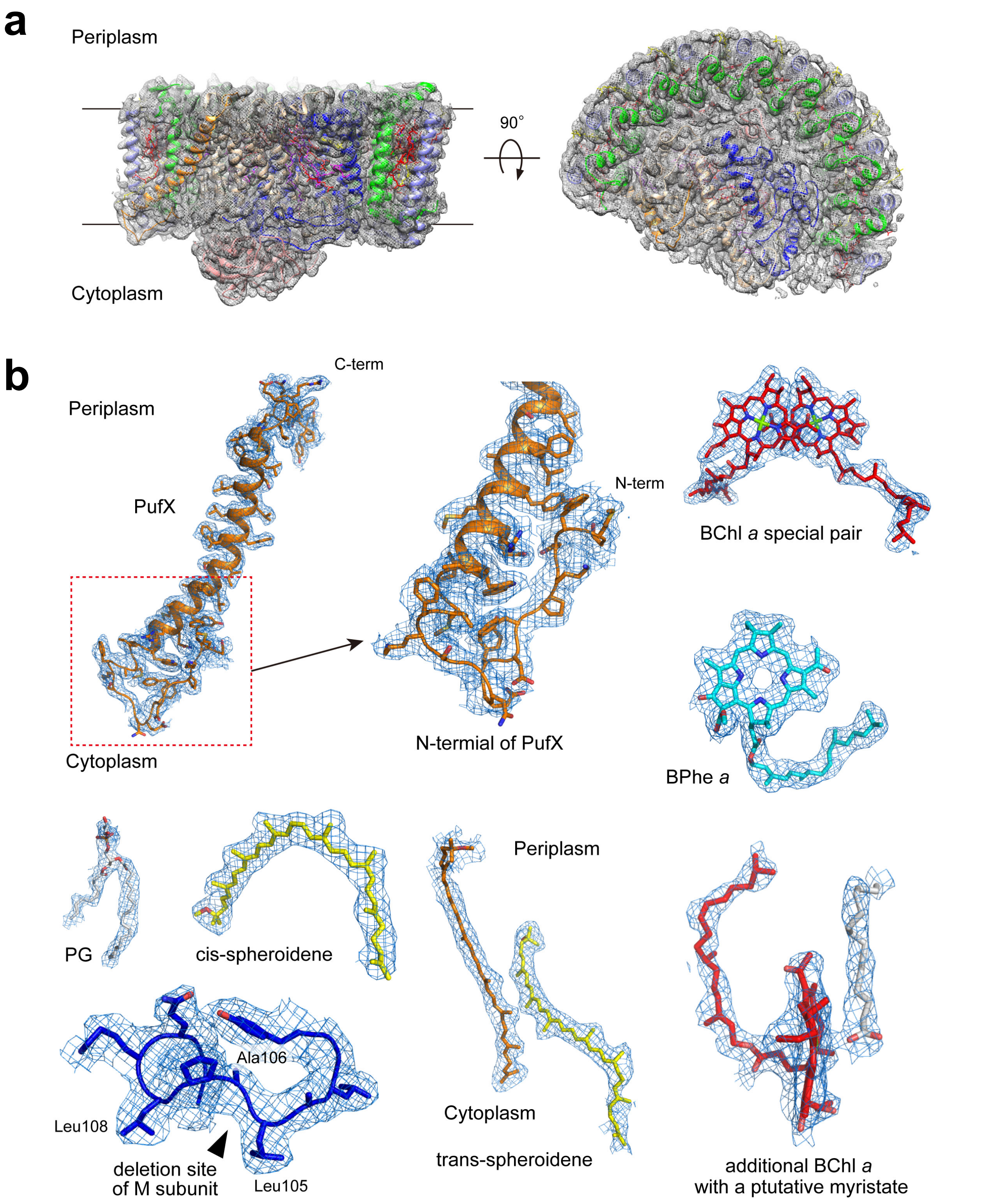

Supplementary Fig. 5 Cryo-EM densities and structural models of the *Rba. capsulatus* LH1–RC complex. (a) Overall structure of the LH1–RC complex. Cartoon representation of the complex with the cryo-EM density in gray mesh. Side view (*left*) parallel to the membrane plane and top view (*right*) from periplasmic side. **(b)** Selected polypeptides and cofactors. The density maps are shown at a contour level of 5.0 σ, except for the additional BChl *a* and a myristate (6.0 σ). Color codes are the same as in the Fig. 1 and 2 of main text.

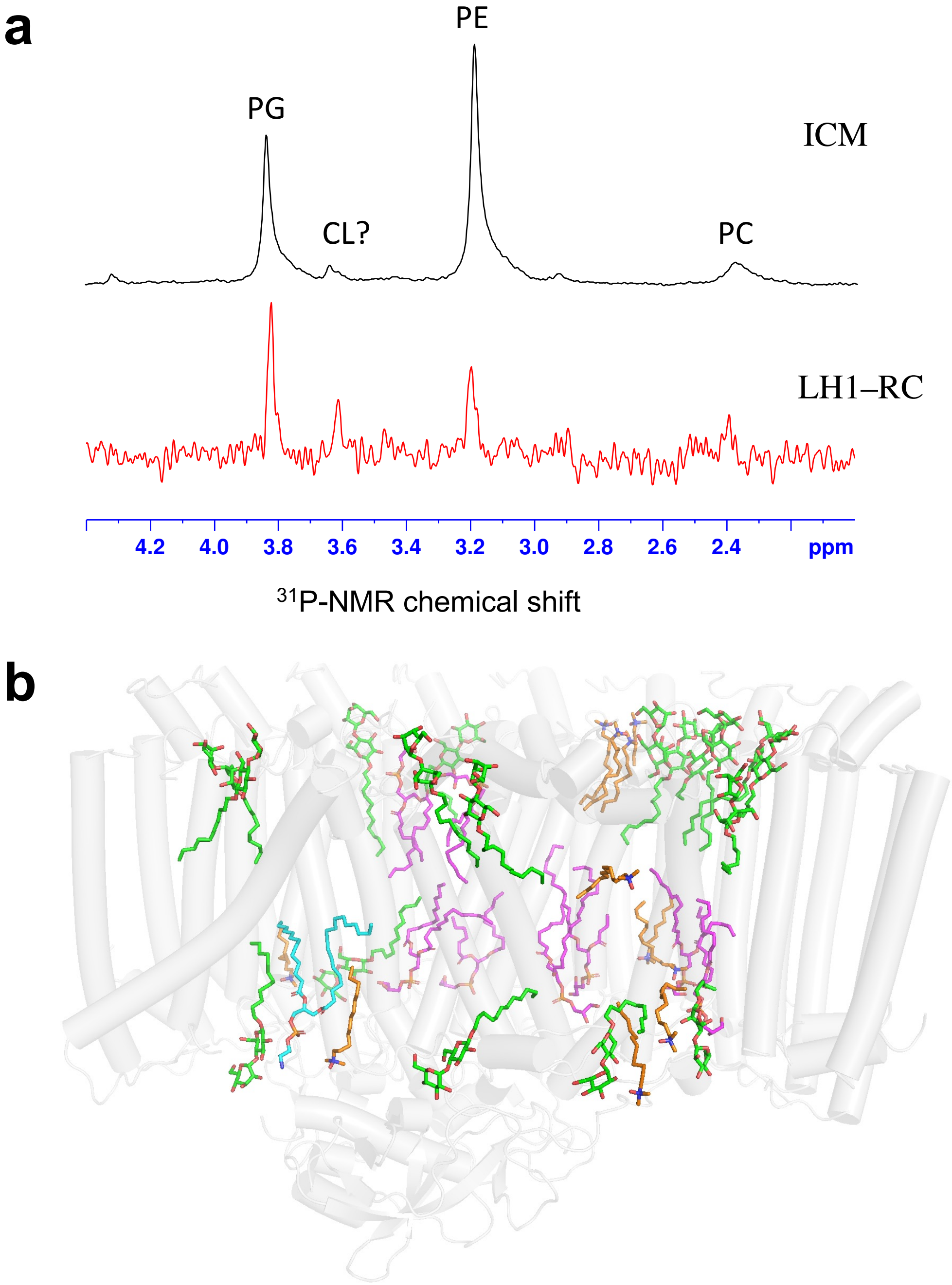

Supplementary Fig. 6 Phospholipids in the *Rba. capsulatus* membranes and LH1–RC. **(a)** ^{31}P-NMR spectra of the phospholipids extracted from chromatophores (ICM) and purified LH1–RC. Phosphatidylglycerol: PG; phosphatidylethanolamine: PE; phosphatidylcholine: PC. The signals near ~3.6 ppm were tentatively assigned to cardiolipin (CL). **(b)** Tentatively assigned phospholipids and detergents in the LH1–RC complex. PG: magenta sticks; PE: cyan sticks; and detergents (DDM: green sticks; LDAO: orange sticks).

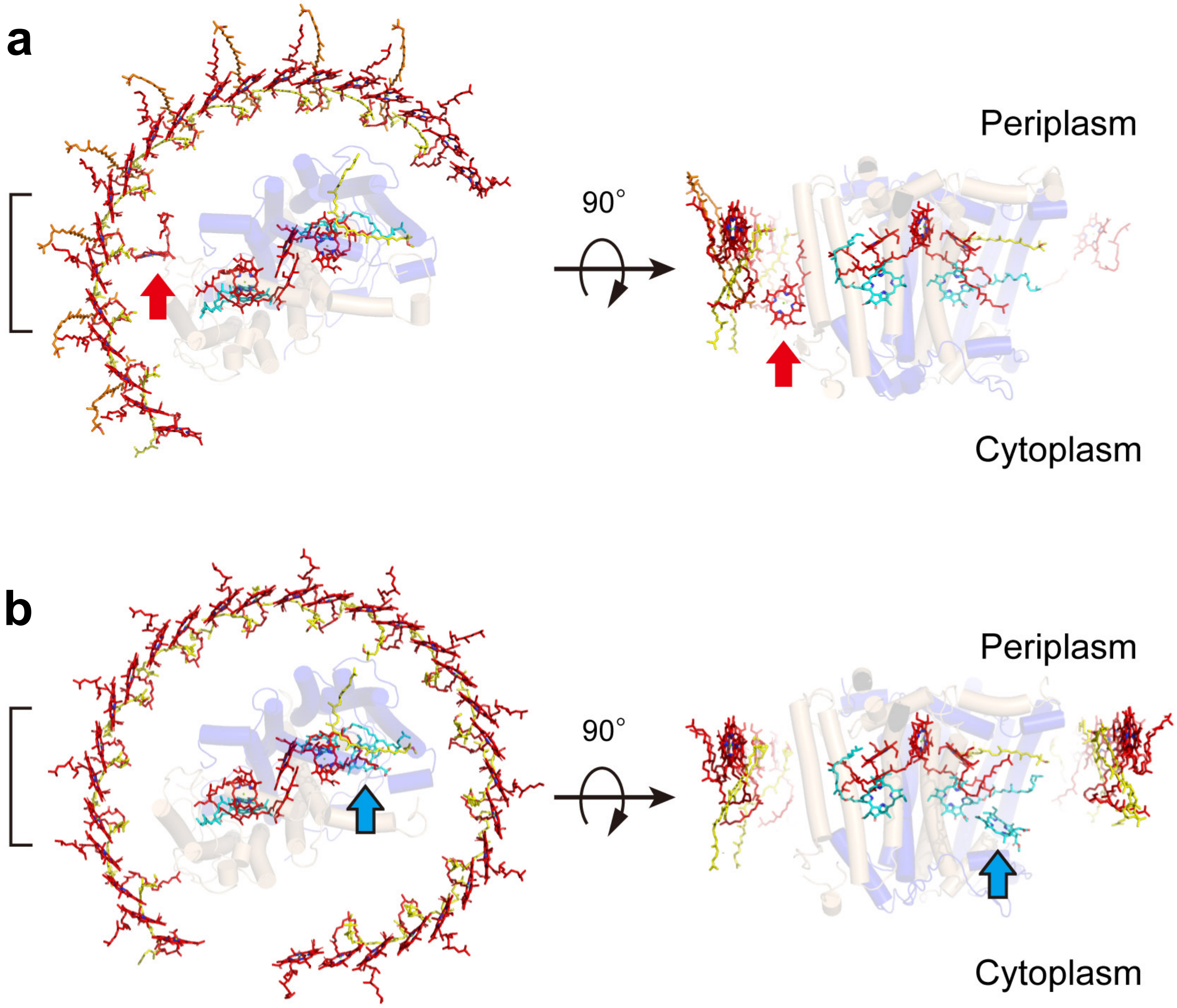

Supplementary Fig. 7 Arrangement comparison of the cofactors in *Rba. capsulatus* and *Rba. veldkampii*. Top view from periplasmic side of the membrane (*left*) and a cross sectional side view along the membrane plane corresponding to the bracket in the top view (*right*) . Color scheme is the same as in Fig. 2 of the main text. For clarity, the L- and M-subunits of the RC are only shown as transparent cartoon representation (L-subunit, wheat; M-subunit, blue). **(a)** for *Rba. capsulatus*. Red arrow indicates an additional BChl *a* near the LH1-α. **(b)** for *Rba. veldkampii*. Blue arrow indicates an additional BPhe *a* near the BPhe associated with the BChl *a* of the special pair.

a

PufX

```
                          10        20        30        40        50        60        70
Rba. capsulatus  MSMFDKPFDYENGS-KFEMGIWIGRQMAYGAFLGSIPFLLGLGLVLGSYGLGLMLPERAHQAPSPYTTEVVVQHATEVV--------
Rba. sphaeroides MADKTIFNDHLNTNPKTNLRLWVAFQMMKGAGWAGGVFFGTLLLIGFFRVVGRMLPIDENPAPAPNITGALETGIELIKHLV-----
Rba. veldkampii  MAEK----HYLDGATKVGMATMGAAAMGKGMGITAVVFFGTVFFVVALAFIGQFLPDRSREAPYPNTIFQVNDIDGTVDGKYTRFAN
                 *:      .: :   *  :    .  *  *    .  *:  : ::     :* :**   . ** *     :      :
```

b

LH1-α

```
Rba. capsulatus   MSKFYKIWLVFDPRRVFVAQGVFLFLLAVLIHLILLSTPAFNWLTVATAKHGYVAAAQ
Rba. sphaeroides  MSKFYKIWMIFDPRRVFVAQGVFLFLLAVMIHLILLSTPSYNWLEISAAKYNRVAVAE
                  ********::*******************:*********::*** :::**:. **.*:
```

LH1-β

```
Rba. capsulatus   MADKNDLSFTGLTDEQAQELHAVYMSGLSAFIAVAVLAHLAVMIWRPWF
Rba. sphaeroides  MADKSDLGYTGLTDEQAQELHSVYMSGLWLFSAVAIVAHLAVYIWRPWF
                  ****.**.:************:******  * ***::***** ******
```

c

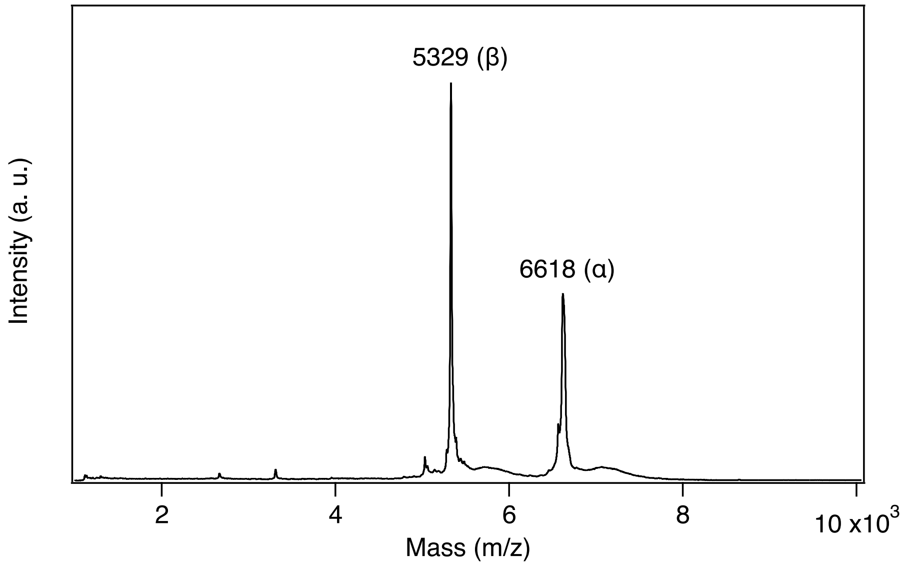

LH1-α (calculated Mw: 6593.9)
MSKFYKIWLVFDPRRVFVAQGVFLFLLAVLIHLILLSTPAFNWLTVATAKHGYVAAAQ

LH1-β (calculated Mw: 5335.1)
ADKNDLSFTGLTDEQAQELHAVYMSGLSAFIAVAVLAHLAVMIWRPWF

PufX (calculated Mw: 7474.5)
SMFDKPFDYENGSKFAMGIWIGRQMAYGAFLGSIPFLFGLGLVLGSYGLGLMLPERAHQAPSPYTTEV

Supplementary Fig. 8 Sequence comparisons and mass spectrum. (a) Sequence alignments of the *Rba. capsulatus* PufX with those in *Rba. sphaeroides* (strain IL106) and *Rba. veldkampii*. Symbol scheme: (*) identical, (.) similar, (:) highly similar. **(b)** Sequence alignments of the *Rba. capsulatus* αβ-polypeptides with those in *Rba. sphaeroides*. **(c)** MALDI-TOF /MS spectrum and predicted amino acid sequences of the purified LH1–RC.

RC-L subunit

(CLUSTAL-W score: 77.7)

```
Rba. capsulatus    MALLSFERKYRVPGGTLIGGSLFDFWVGPFYVGFFGVTTIFFATLGFLLILWGAAMQGTW 60
Rba. sphaeroides   MALLSFERKYRVPGGTLVGGNLFDFWVGPFYVGFFGVATFFFAALGIILIAWSAVLQGTW
                   ****************:**.****************:*:***:**::** *.*.:****

                   NPQLISIFPPPVENGLNVAALDKGGLWQVITVCATGAFCSWALREVEICRKLGIGFHIPV 120
                   NPQLISVYPPALEYGLGGAPLAKGGLWQIITICATGAFVSWALREVEICRKLGIGYHIPF
                   ******::**.:* **. *.* ******:**:****** ****************:***.

                   AFSMAIFAYLVLVVIRPMMMGSWGYAFPYGIWTHLDWVSNTGYTYGNFHYNPFHMLGISL 180
                   AFAFAILAYLTLVLFRPVMMGAWGYAFPYGIWTHLDWVSNTGYTYGNFHYNPAHMIAISF
                   **::**:***.**::**:***:****************************** **:.**:

                   FFTTAWALAMHGALVLSAANPVKGKTMRTPDHEDTYFRDLMGYSVGTLGIHRLGLLLALN 240
                   FFTNALALALHGALVLSAANPEKGKEMRTPDHEDTFFRDLVGYSIGTLGIHRLGLLLSLS
                   ***.* ***:*********** *** *********:****:***:************:*.

                   AVFWSAMCMLVSGTIYFDLWSDWWYWWVNMPFWVDMAGGING
                   AVFFSALCMIITGTIWFDQWVDWWQWWVKLPWWANIPGGING
                   ***:**:**::***:** * *** ***::*:*.::.*****
```

RC-M subunit

(CLUSTAL-W score: 74.9)

```
Rba. capsulatus    MAEYQNFFNQVQVAGAPEMGLKEDVDTYERTPAG-MLSILGWMGNAQIGPIYLGIAGTVS
Rba. sphaeroides   MAEYQNIFSQVQVRGPADLGMTEDVNLANRSGVGPFSTLLGWFGNAQLGPIYLGSLGVLS
                   ******:*.**** *..::*:.***:  :*: .* : ::***:****:******  *.:*

                   LVFGAAWFFTIGVWYWYQAGFDPFIFMRDLFFFSLEPPPAEYGLAL-APLKQGGVWQIAS
                   LFSGLMWFFTIGIWFWYQAGWNPAVFLRDLFFFSLEPPAPEYGLSFAAPLKEGGLWLIAS
                   *. *  ******:*:*****::* :*:***********..*****: ****:**:* ***

                   LFMAISVIAWWVRVYTRADQLGMGKHMAWAFLSAIWLWSVLGFWRPILMGSWSVGVPYGI
                   FFMFVAVWSWWGRTYLRAQALGMGKHTAWAFLSAIWLWMVLGFIRPILMGSWSEAVPYGI
                   :** ::* :** *.* **: ****** *********** **** ********* .*****

                   FSHLDWTNQFSLDHGNLFYNPFHGLSIAALYGSALLFAMHGATILAVTRFGGERELEQIA
                   FSHLDWTNNFSLVHGNLFYNPFHGLSIAFLYGSALLFAMHGATILAVSRFGGERELEQIA
                   ********:*** *************** ******************:************

                   DRGTASERAALFWRWTMGFNATMEGIHRWAIWMAVMVTLTGGIGILLSGTVVDNWYVWAQ
                   DRGTAAERAALFWRWTMGFNATMEGIHRWAIWMAVLVTLTGGIGILLSGTVVDNWYVWGQ
                   *****:*****************************:**********************.*
                   VHGYAPVTP
                   NHGMAPLN-
                    ** **:.
```

105 106

L-APLK

FAAPLK

RC-H subunit

(CLUSTAL-W score: 60.5)

```
Rba. capsulatus    MVGVNFFGDFDLASLAIWSFWAFLAYLIYYLQTENMREGYPLENDDGLPSANQGPFPVPS
Rba. sphaeroides   MVGVTAFGNFDLASLAIYSFWIFLAGLIYYLQTENMREGYPLENEDGTPAANQGPFPLPK
                   ****. **:********:*** *** *******************:** *:*******:*.

                   PKTFELADGRKIVVPSVENEEAHRRTDLALERTSVNEGYPFRPTGNPMLDGVGPASWVPR
                   PKTFILPHGRGTLTVPGPESEDR---PIALARTAVSEGFPHAPTGDPMKDGVGPASWVAR
                   **** *..**  :.  .  :.* :     :** **:*.**:*. ***:** *********.*

                   RDEPEVDAHGHNKIQPMR-KTEMTVSAGRDPRGMPVQAGDTEVVGKIVDMWVDIPEQLVR
                   RDLPELDGHGHNKIKPMKAAAGFHVSAGKNPIGLPVRGCDLEIAGKVVDIWVDIPEQMAR
                   ** **:*.******:**:   : : ****::* *:**:. * *:.**:**:*******:.*

                   YLEVELNSGKKKLLPMTMLKIWSDRVRVNAITSDLFDTIPDIKSPDVVTKLEEDKISAYV
                   FLEVELKDGSTRLLPMQMVKVQSNRVHVNALSSDLFAGIPTIKSPTEVTLLEEDKICGYV
                   :*****:.*..:**** *:*: *:**:***::****  ** ****  ** ******..**

                   AGGYMYAKGVKPYA---------
                   AGGLMYAAPKRKSVVAAMLAEYA
                   *** ***    :  .
```

Supplementary Fig. 9 Sequence comparisons of the RC proteins between *Rba. capsulatus* and *Rba. sphaeroides*. His residues coordinating to the special pair BChls *a* are shown in red, bold fonts. Typical sequence differences between the two species are shown by blue boxes. Symbol scheme: (*) identical, (.) similar, (:) highly similar.

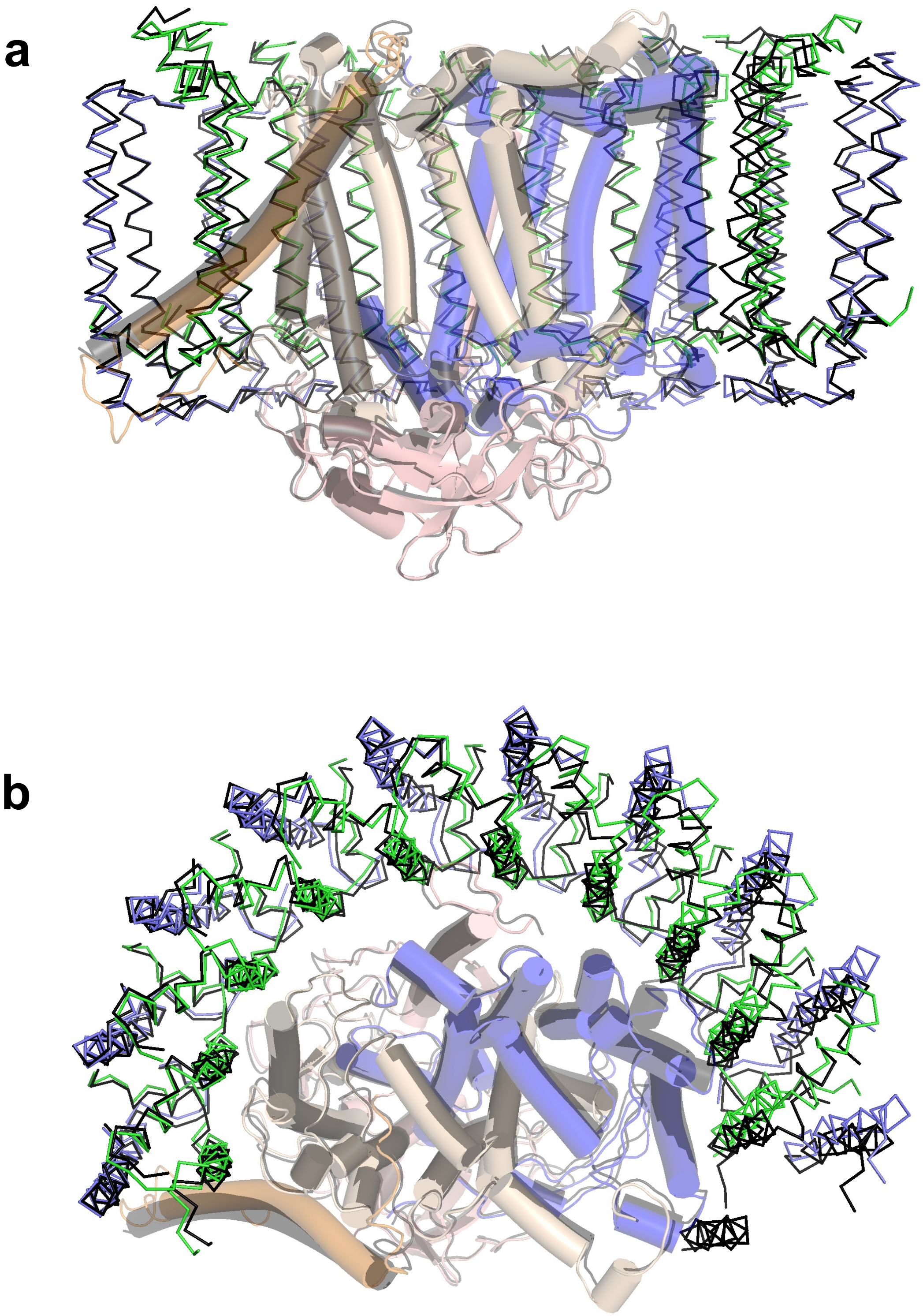

Supplementary Fig. 10 Structural comparison of the *Rba. capsulatus* LH1–RC with the protein-U-deleted LH1–RC from a *Rba. sphaeroides* ΔU mutant strain. **(a)** Side view of mainchain superposition of the *Rba. capsulatus* LH1–RC (colored) with the ΔU LH1–RC (PDB: 7VY3, black ribbons for LH1 and gray cartoons for the RC). **(b)** Top view of **(a)** from periplasmic side. All cofactors are omitted for clarity.

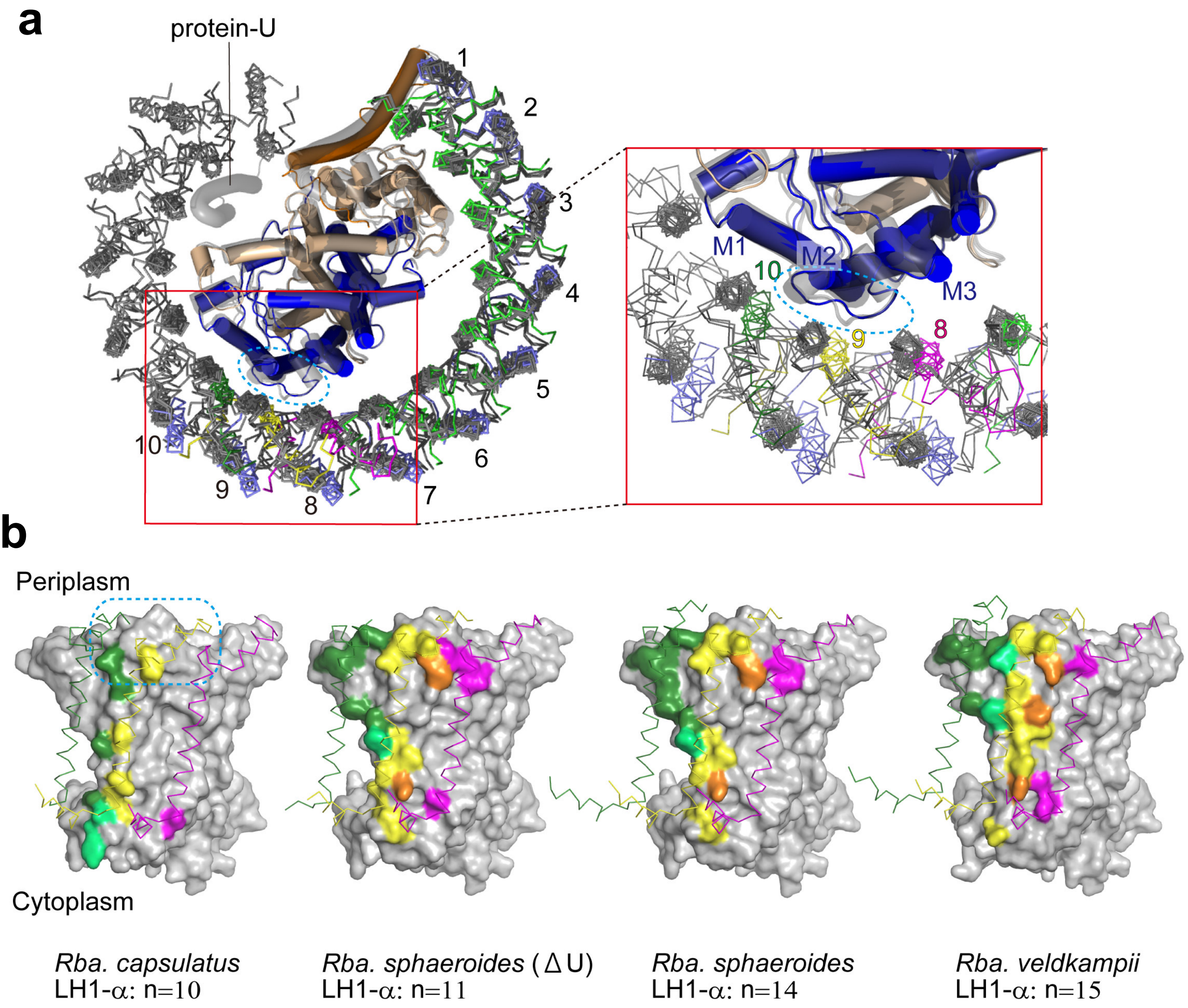

Supplementary Fig. 11 Structural comparison of the *Rba. capsulatus* LH1–RC with the LH1–RCs from *Rba. sphaeroides*, *Rba. sphaeroides* ΔU mutant strain, and *Rba. veldkampii*. **(a)** Top view of mainchain superposition of the *Rba. capsulatus* LH1–RC (colored) with the LH1–RCs from *Rba. sphaeroides* , *Rba. sphaeroides* ΔU, *Rba. veldkampii* (PDB: 7F0L, 7VY3, 7DDQ, respectively. Gray ribbons for LH1 and gray cartoons for the RC and protein-U). The LH1-α of 8th, 9th, and 10th associating with the RC directly are shown in magenta, yellow, and forest green. **(b)** Surface representations of the RC-M subunits with the LH1-α 8th, 9th, and 10th polypeptides shown in ribbon representation. M-subunit residues interacting with the LH1-α of 8th, 9th, 10th, both 8th and 9th, or both 9th and 10th are colored in magenta, yellow, forest green, orange, or lime green, respectively. Dashed cyan oval indicates the loop region between helices M1 and M2 of M-subunit.
